# Evaluation of different scoring systems for spinal metastases based on a Chinese cohort

**DOI:** 10.1002/cam4.5272

**Published:** 2022-09-21

**Authors:** Zhehuang Li, Liangyu Guo, Bairu Guo, Peng Zhang, Jiaqiang Wang, Xin Wang, Weitao Yao

**Affiliations:** ^1^ Department of Musculoskeletal Oncology Affiliated Cancer Hospital of Zhengzhou University, Henan Cancer Hospital Zhengzhou China

**Keywords:** prediction, prognosis, spine metastases, surgery, survival

## Abstract

**Introductions:**

The spine is one of the most common sites of metastasis for malignancies. This study aimed to compare the predictive performance of seven commonly used prognostic scoring systems for surgically treated spine metastases. It is expected to assist surgeons in selecting appropriate scoring systems to support clinical decision‐making and better inform patients.

**Methods:**

We performed a retrospective study involving 268 surgically treated patients with spine metastases between 2017 and 2020 at a single regional oncology center in China. The revised Tokuhashi, Tomita, modified Bauer, revised Katagiri, van der Linden, Skeletal Oncology Research Group (SORG) nomogram, and SORG machine‐learning (ML) scoring systems were externally validated. The area under the curve (AUC) of the receiver operating characteristic curve was used to evaluate sensitivity and specificity at different postoperative time points. The actual survival time was compared with the reference survival time provided in the original publication.

**Results:**

In the present study, the median survival was 16.6 months. The SORG ML scoring system demonstrated the highest accuracy in predicting 90‐day (AUC: 0.743) and 1‐year survival (AUC: 0.787). The revised Katagiri demonstrated the highest accuracy (AUC: 0.761) in predicting 180‐day survival. The revised Katagiri demonstrated the highest accuracy (AUC: 0.779) in predicting 2‐year survival. Based on this series, the actual life expectancy was underestimated compared with the original reference survival time.

**Conclusions:**

None of the scoring systems can perform optimally at all time points and for all pathology types, and the reference survival times provided in the original study need to be updated. A cautious awareness of the underestimation by these models is of paramount importance in relation to current patients.

## 1 INTRODUCTION

The spine is one of the most common sites of metastasis for malignancies, other than the lung and liver. In patients with systemic cancer, approximately 60%–70% develop spinal metastases, and approximately 10% are symptomatic.^1^, ^2^ Surgery potentially improves quality of life for indicated patients.^3^, ^4^, ^5^ However, the risk of surgery‐related complications, treatment cost, and surgical benefit require careful evaluation.

Expected survival has long been considered a strongly weighted metric for determining surgical candidacy in patients with spinal metastases. A scoring system is a composite of multiple prognostic factors that have been weighted and potentially provide individual survival predictions for a given patient as well as treatment recommendations. Some of the most common predictive scoring systems include the revised Tokuhashi,^6^ Tomita,^7^ modified Bauer,^8^ revised Katagiri,^9^ and van der Linden (VDL) scoring systems.^10^ These classic scoring systems are widely used and commonly accepted. Notwithstanding, it must be noted that the sample sizes were small, studies on these scoring systems were published several years back, and the treatments and outcomes for malignancies have evolved significantly since then. In recent years, the Skeletal Oncology Research Group (SORG) nomogram,^11^ and machine learning (ML)^12^ scoring systems have also been proposed and proven to be ideally accurate.^13^, ^14^, ^15^

In this study, which involved 268 surgically treated patients with metastatic spine disease at a single regional oncology center in China, we compared the predictive performance of seven scoring systems at various time points. This study is expected to assist surgeons in selecting appropriate scoring systems to support clinical decision‐making and better inform patients. Although previous studies have compared different scoring systems,^8^, ^16^, ^17^, ^18^ the present study has certain unique implications. First, we compared the traditional classic scoring systems with a novel, advanced scoring systems (e.g., the SORG nomogram and SORG ML scoring system), a phenomenon that has rarely been investigated. Second, our study used a more recent and concentrated cohort, implying that the findings and recommendations emerging from this study are more relevant to current clinical practice. Third, studies evaluating different scoring systems based on Asian populations are lacking.

## 2 METHODS

### 2.1 Study design and subject inclusion

We retrospectively reviewed the charts of all patients who underwent surgery for spinal metastases at our institution between January 2017 and August 2020. Our institutional review board approved a waiver of our study due to its retrospective nature.

The therapeutic approaches were managed by a multidisciplinary team, and the decision to perform surgery was based on the patient's medical fitness, clinical presentation (neurologic deficit, spinal instability, and intractable pain), oncological status, and feasibility of surgical treatment. Patient follow‐ups were conducted prospectively by the LinkDoc company under the authority of the hospital. Postoperatively, follow‐up evaluations were scheduled 3, 6, and 12 months after the first year; every 6 months for the subsequent 2 years; and annually thereafter.

The inclusion criteria were as follows: (1) patient age > 18 years when surgery was performed, (2) pathologically confirmed diagnosis of spinal metastasis, and (3) full accessibility of data required for scoring‐system‐based survival assessment from the patient's electronic medical record.

The exclusion criteria were as follows: (1) loss to follow‐up within 1 year after surgery without the capacity to definitively determine survival at the 1‐year time point and (2) percutaneous vertebroplasty as the exclusive surgical procedure.

### 2.2 Scoring systems

The seven scoring systems we evaluated in the present study can be categorized into traditional and advanced scoring systems.^19^ Traditional scoring systems, including the revised Tokuhashi,^6^ Tomita,^7^ modified Bauer,^8^ revised Katagiri,^9^ and VDL were developed by assigning values to predictive factors according to the effect estimates (e.g., hazard ratios), and the scores of individual factors are summed to obtain the total score. Based on their total scores, patients were stratified into different subgroups with varying prognoses and treatment recommendations. Regarding advanced scoring systems, including the SORG nomogram^19^ and SORG ML scoring system,^12^ the final output is an individualized survival probability.

To obtain the survival probability on the SORG nomogram, each factor is assigned its own point on the corresponding axis, with a vertical line drawn downward to indicate the number of points. A vertical line can be drawn downward from the point sum on the “total points” axis; thus, the 90‐day and 1‐year survival probabilities can be measured.

To obtain the survival probability on the SORG ML scoring system, the clinical characteristics of individual patients were input on an open access web application (https://sorg‐apps.shinyapps.io/spinemetssurvival/). The 90‐day and 1‐year survival predictions were provided on the tabs.

### 2.3 Statistical analysis

The receiver operating characteristic (ROC) curves for each scoring system at different time points (90 days, 180 days, 1 year, and 2 years after surgery) were constructed by plotting the true‐positive (sensitivity) and false‐positive (1—specificity) rates. The area under the curve (AUC) was computed for each ROC. The AUC results were considered outstanding for AUC values exceeding 0.9, excellent for AUC values between 0.8 and 0.9, acceptable for AUC values between 0.7 and 0.8, and poor for AUC values <0.7. Kaplan–Meier survival estimates were calculated for different patient subgroups as described in the original studies. The log‐rank test was used to compare the survival distributions of the different subgroups. Regarding advanced scoring systems, the SORG nomogram and SORG ML scoring system, which yield an individualized survival probability for a specific patient, calibration curve, slope, and intercept were also used to evaluate calibration. The slope evaluates the spread of the estimated risks and has a target value of 1. A slope <1 suggests that estimated risks are too high for patients at high risk and too low for patients at low risk. A slope >1 suggests the contrary. The intercept reflects the “calibration‐in‐the‐large,” with a target value of 0. A negative intercept suggests overestimation, whereas a positive intercept suggests underestimation.^20^ The Brier score is the average squared difference between predicted risk and observed risk, ranging from 0 (perfect) to 1 (worst); it was used for overall performance evaluation. The null‐model Brier score simply predicts the overall prevalence of the outcome in the validation. A two‐tailed *p* value <0.05 was considered statistically significant.^21^ We used R version 4.0.3 for Windows (R Project for Statistical Computing, http://www.r‐project.org/) for statistical analysis.

## 3 RESULTS

### 3.1 Patient demographics and primary tumor

Based on the inclusion and exclusion criteria, 268 patients were included in the present study. There were 124 male and 144 female patients with an average age of 55.4 ± 10.6 years (range, 23–78 years) at the time of surgery. The mean body mass index was 23.7 ± 3.6 kg/m^2^. The median Karnofsky Performance Scale score and Eastern Cooperative Oncology Group performance status were 60 and 3, respectively. Neurological deficits were present in 41.8% of patients, preoperatively. Five patents (1.9%) had American Spinal Injury Association (ASIA) Impairment Scale A, 16 (6.0%) ASIA B, 30 (11.2%) ASIA C, and 61 (22.8%) ASIA D. A total of 153 (57.1%) tumors were located in the thoracic spine, 69 (25.7%) in the lumbar spine, 24 (9.0%) in the cervical spine, and 22 (8.2%) spanning multiple regions. The baseline characteristics of the patients are summarized in Table 1.

**TABLE 1:** Baseline characteristics.

Characteristic	Value
Demographic	
Age (years)	55.4 ± 10.6
Male sex	124 (46.3%)
Clinical and surgical	
Preoperative ASIA impairment scale	
A to C	51 (19.0%)
D or E	217 (81.0%)
ECOG performance status	
Score 0–2 (≤50% of waking hours bed or chair bound)	103 (38.5%)
Score 3–4 (>50% of waking hours bed or chair bound)	165 (61.5%)
Surgery	
Corpectomy or vertebrectomy with stabilization	117 (43.7%)
Decompression and stabilization	70 (26.1%)
Decompression alone	80 (29.9%)
Stabilization alone	1 (0.4%)
Oncologic status	
No. of spine metastases	
1 level	91 (34.0%)
2 levels	35 (13.1%)
≥3 levels	142 (53.0%)
Visceral metastases at time of surgery	
None	148 (55.2%)
Liver or lung	99 (36.9%)
Brain	30 (11.2%)
Prior local radiotherapy	26 (9.7%)
Previous systemic therapy	147 (54.9%)
Laboratory data	
WBC count (×10^3^/μl)	6.73 ± 3.04
Hemoglobin (g/dl)	12.3 ± 1.8
Platelet count (×10^3^/μl)	226.6 ± 76.8
Neutrophil count (×10^3^/μl)	4.86 ± 2.58
Lymphocyte count (×10^3^/μl)	1.38 ± 0.71
Albumin (g/dl)	4.0 ± 0.5
Alkaline phosphatase (IU/L)	124.0 ± 148.6

Abbreviations: ASIA, American Spinal Injury Association; BMI, body mass index; ECOG, Eastern Cooperative Oncology Group.

The primary tumor histology was as follows: lung (*n* = 71, 26.5%), breast (*n* = 52, 19.4%), multiple myeloma (*n* = 21, 7.8%), liver (*n* = 15, 5.6%), and other (*n* = 109, 40.7%). The primary tumor types and their survival are summarized in Table 2.

**TABLE 2:** Primary tumor types and survival.

Primary tumor	No. of patients	Mean survival days from surgery	Median survival days from surgery
Breast	52 (19.4%)	665	1023
Colon	5 (1.9%)	231	226
Esophagus	4 (1.5%)	166	130
Stomach	5 (1.9%)	275	324
Kidney	11 (4.1%)	442	407
Liver	15 (5.6%)	350	183
Lung	71 (26.5%)	362	291
Leukemia	1 (0.4%)	104	104
Lymphoma	6 (2.2%)	484	NR^a^
Mediastinum	1 (0.4%)	1369	NR
Melanoma	1 (0.4%)	677	677
Multiple myeloma	21 (7.8%)	601	NR
Nasopharynx	4 (1.5%)	288	209
Ovary	1 (0.4%)	732	NR
Pancreas	2 (0.7%)	257	257
Prostate	13 (4.9%)	501	NR
Rectum	10 (3.7)	256	264
Sarcoma	7 (2.6%)	712	NR
Testicle	1 (0.4%)	48	48
Thyroid	12 (4.5%)	658	NR
Unknown primary tumor	11 (4.1%)	535	460
Urinary tract	2 (0.8%)	144	144
Uterus	12 (4.5%)	431	345

^a^ NR, median survival was not reached.

### 3.2 Treatment

A total of 147 (54.9%) patients received preoperative systematic therapy. Preoperative radiotherapy was performed in 26 (9.7%) patients. The surgeries were all performed under general anesthesia in a single stage. Surgeries lasted an average time of 241.9 ± 101.6 min, with an average blood loss of 1081.0 ± 1008.3 ml. The posterior approach was employed in 241 (89.9%) cases, the anterior approach in 20 (7.5%), and the combined approach in 7 (2.6%). Corpectomy or vertebrectomy with stabilization was performed in 117 (43.7%) patients, decompression and stabilization in 70 (26.1%), decompression alone in 80 (29.9%), and stabilization alone in one (0.4%).

### 3.3 Overall predictive accuracy of survival in the seven scoring systems

In all 268 patients, the median survival was 16.6 months. The 3‐month, 6‐month, 1‐year, and 2‐year overall survival rates were 86.6%, 71.3%, 55.2%, and 39.8% respectively.

**FIGURE 1:**
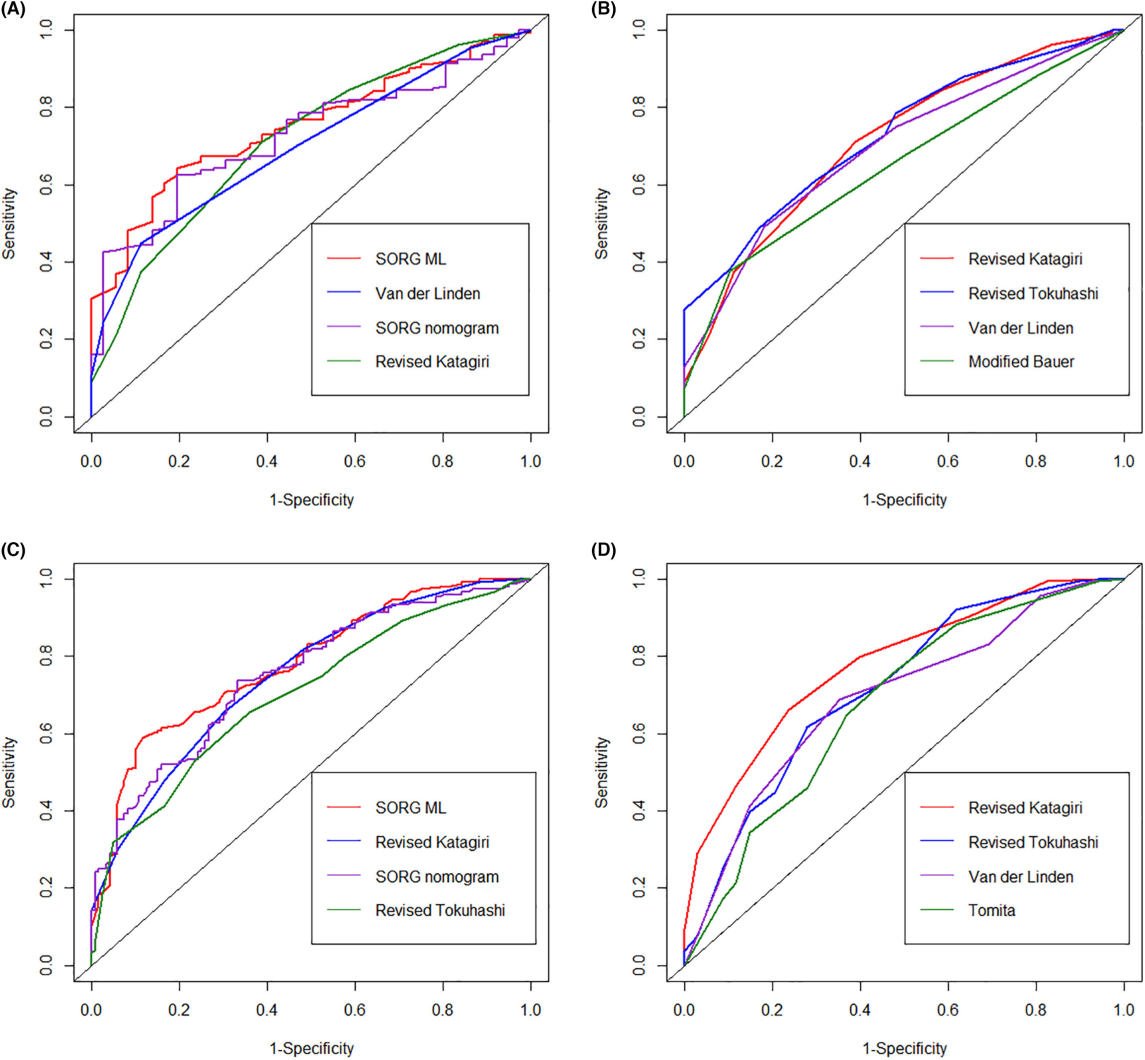
The ROC curves for 90‐day (A), 180‐day (B), 1‐year (C), and 2‐year (D) survival prediction of the scoring systems. Only scoring systems with the top four AUC values are shown.

Regarding the prediction of short‐term survival, which was defined as 90‐day survival after the index surgery in the present study, the SORG ML scoring system demonstrated the highest accuracy (AUC: 0.743). The revised Katagiri (AUC: 0.711), VDL (AUC: 0.725), and SORG nomogram (AUC: 0.722) also exhibited an acceptable performance in predicting 90‐day survival (Table 3, Figure 1).

**TABLE 3:** Area under curve (AUC) from receiver operating characteristic (ROC) curve for 90‐day, 180‐day, 1‐year, and 2‐year survival after the surgery.

Scoring system	All patients	Breast	Lung
90‐day survival			
Tomita	0.595 (0.494–0.687)	0.824 (NA^a^)	0.465 (0.280–0.649)
Revised Tokuhashi	0.650 (0.548–0.745)	0.922 (NA)	0.621 (0.437–0.805)
Modified Bauer	0.618 (0.518–0.708)	0.765 (NA)	0.478 (0.323–0.633)
Revised Katagiri	0.711 (0.624–0.801)	0.931 (NA)	0.623 (0.456–0.789)
van der Linden	0.725 (0.634–0.797)	0.951 (NA)	0.556 (0.403–0.710)
SORG Nomogram	0.722 (0.645–0.790)	0.765 (NA)	0.617 (0.440–0.794)
SORG ML	0.743 (0.666–0.817)	0.804 (NA)	0.665 (0.504–0.801)
180‐day survival			
Tomita	0.636 (0.564–0.706)	0.794 (0.673–0.915)	0.440 (0.301–0.578)
Revised Tokuhashi	0.723 (0.653–0.785)	0.851 (0.654–1.000)	0.596 (0.459–0.733)
Modified Bauer	0.671 (0.602–0.739)	0.787 (0.716–0.859)	0.511 (0.382–0.640)
Revised Katagiri	0.761 (0.696–0.826)	0.860 (0.669–1.000)	0.676 (0.548–0.803)
van der Linden	0.698 (0.624–0.769)	0.866 (0.733–0.999)	0.603 (0.481–0.726)
1‐year survival			
Tomita	0.620 (0.552–0.689)	0.566 (0.389–0.743)	0.413 (0.283–0.543)
Revised Tokuhashi	0.702 (0.637–0.767)	0.644 (0.449–0.839)	0.531 (0.396–0.667)
Modified Bauer	0.646 (0.575–0.708)	0.565 (0.392–0.737)	0.446 (0.325–0.567)
Revised Katagiri	0.759 (0.698–0.814)	0.702 (0.521–0.883)	0.660 (0.534–0.785)
van der Linden	0.671 (0.605–0.738)	0.691 (0.513–0.869)	0.567 (0.439–0.695)
SORG Nomogram	0.757 (0.696–0.811)	0.769 (0.628–0.910)	0.623 (0.490–0.757)
SORG ML	0.787 (0.730–0.838)	0.740 (0.583–0.886)	0.793 (0.679–0.879)
2‐year survival			
Tomita	0.679 (0.639–0.719)	0.631 (0.548–0.715)	0.391 (0.300–0.484)
Revised Tokuhashi	0.717 (0.679–0.755)	0.618 (0.530–0.706)	0.439 (0.351–0.527)
Modified Bauer	0.678 (0.639–0.717)	0.620 (0.541–0.700)	0.378 (0.301–0.455)
Revised Katagiri	0.779 (0.747–0.811)	0.649 (0.563–0.735)	0.543 (0.458–0.627)
van der Linden	0.694 (0.657–0.731)	0.654 (0.573–0.735)	0.531 (0.448–0.614)

*Note*: The SORG nomogram and SORG Machine‐learning do not provide a function for 180‐day and 2‐year survival prediction, so validations of these time points were not performed.

Abbreviation: ML, Machine‐learning; SORG, Skeletal Oncology Research Group.

^a^ NA: not available to calculate the 95% confidence interval.

In terms of predicting mid‐term (180‐day) postoperative survival, the revised Katagiri demonstrated the highest accuracy (AUC: 0.761), followed by the revised Tokuhashi (AUC: 0.723, Table 3, Figure 1). The SORG nomogram and SORG ML scoring system do not provide a function for 180‐day survival prediction; hence, validation of these tools was not performed.

With regard to predicting long‐term (1‐year) postoperative survival, the SORG ML scoring system demonstrated the highest accuracy (AUC: 0.787), followed by the revised Katagiri (AUC: 0.759), SORG nomogram (AUC: 0.757), and revised Tokuhashi (AUC: 0.702), which also exhibited an acceptable performance in predicting 1‐year survival (Table 3, Figure 1).

For 2‐year postoperative survival prediction, the revised Tokuhashi (AUC: 0.717) and revised Katagiri (AUC: 0.779) demonstrated acceptable performance.

### 3.4 Predictive accuracy of survival: Breast cancer

Regarding the prediction of 90‐day survival in patients with metastatic breast cancer of the spine, the VDL scoring system was the most accurate (AUC: 0.951). The revised Katagiri (AUC: 0.931) and revised Tokuhashi (AUC: 0.922) all exhibited outstanding accuracy in predicting 90‐day survival for breast cancer (Table 3). However, only one patient with breast cancer passed away within 90 days after surgery; therefore, the 90‐day survival prediction should be cautiously evaluated. With regard to predicting 180‐day survival, the VDL scoring system demonstrated the highest accuracy (AUC: 0.866). The revised Katagiri (AUC: 0.860) and revised Tokuhashi (AUC: 0.851) also exhibited excellent accuracy (Table 3). In terms of predicting 1‐year survival, the SORG nomogram demonstrated the highest accuracy (AUC: 0.769). The SORG ML (AUC: 0.740) and revised Katagiri (AUC: 0.702) also exhibited acceptable accuracy (Table 3).

### 3.5 Predictive accuracy of survival: lung cancer

Regarding the prediction of 90‐day survival in patients with metastatic lung cancer of the spine, the SORG ML scoring system was the most accurate (AUC: 0.665), yet not ideal. None of the scoring systems yielded an AUC > 0.7 (Table 3). In terms of predicting 180‐day survival, the revised Katagiri scoring system demonstrated the highest accuracy (AUC: 0.676). None of the scoring systems yielded an AUC > 0.7 (Table 3). The SORG ML scoring system demonstrated the highest accuracy (AUC: 0.793) in predicting 1‐year survival. Otherwise, no other scoring system yielded an AUC > 0.7 (Table 3).

### 3.6 Comparison of reference survival model with actual survival

The mean, median, 90‐day, 180‐day, 1‐year, and 2‐year survivals of each subgroup in relation to the scoring systems are summarized in Table 4.

**TABLE 4:** Actual survival time of each scoring systems.

Scoring system (points)	No.	Mean survival days	Median survival days	90‐day survival	180‐day survival	1‐year survival	2‐year survival	Hazard ratio	*p*
Tomita									<0.001*
Long‐term (2, 3)	56	649	NA	94.6%	89.3%	80.4%	64.6%	R	R
Mid‐term (4, 5)	64	484	621	89.1%	75.0%	53.1%	42.2%	1.96 (1.15–3.35)	0.024*
Short‐term (6, 7)	75	445	354	81.3%	65.3%	49.3%	34.2%	2.48 (1.49–4.13)	0.001*
Palliative care (8–10)	73	377	291	83.6%	60.3%	43.8%	23.0%	3.12 (1.88–5.18)	<0.001*
Revised Tokuhashi									<0.001*
No surgery (0–8)	162	395	266	82.7%	60.5%	43.2%	28.3%	4.44 (2.17–9.10)	<0.001*
Palliative (9–11)	76	566	721	89.5%	82.9%	67.1%	49.5%	2.28 (1.06–4.89)	<0.027*
Excisional (12–15)	30	706	NA	100%	100%	90.0%	74.2%	R	R
Modified Bauer									<0.001*
Supportive care (0–1)	99	382	291	79.8%	61.6%	44.4%	24.7%	2.71 (1.80–4.10)	<0.001*
Short‐term palliation (2)	89	455	354	88.8%	65.2%	49.4%	34.8%	2.02 (1.31–3.11)	<0.001*
Mid‐term local control (3, 4)	80	625	1023	92.5%	90.0%	75.0%	61.6%	R	R
Revised Katagiri									<0.001*
Low‐risk (0–3)	91	651	NA	95.6%	90.1%	78.0%	64.7%	R	R
Intermediate‐risk (4–6)	126	455	400	86.5%	69.8%	52.4%	34.2%	2.53 (1.69–3.79)	<0.001*
High‐risk (7–10)	51	228	129	70.6%	41.2%	21.6%	4.8%	6.16 (3.90–9.75)	<0.001*
van der Linden									0.004*
A (0–3)	210	433	364	83.3%	66.2%	83.3%	35.9%	3.00 (0.95–9.43)	0.292
B (4, 5)	50	608	721	98.0%	88.0%	98.0%	48.3%	1.64 (0.49–5.45)	0.082
C (6)	8	864	1023	100.0%	100.0%	100.0%	75.0%	R	R

Abbreviations: R, reference; SORG, Skeletal Oncology Research Group.

* *p* < 0.05.

In the original publication by Tomita,^7^ the mean survival periods were 49.9, 23.5, 15.0, and 5.9 months in patients with 2–3, 4–5, 6–7, and 8–10 points, respectively. In the present study, the mean survival periods were 21.3 months (649 days), 15.9 months (484 days), 14.6 months (445 days), and 12.4 months (377 days) in patients with 2–3, 4–5, 6–7, and 8–10 points, respectively.

In the original publication of the revised Tokuhashi scoring system,^6^ 85.3% of patients survived <6 months, with 0–8 points; 73.1% survived >6 months, with 9–11 points; and 95.4% survived >1 year, with 12–15 points. In the present study, 39.5% of patients survived <6 months, with 0–8 points; 82.9% survived >6 months, with 9–11 points; and 90% survived >1 year, with 12–15 points.

In the original publication of the modified Bauer scoring system,^8^ the median survival was 4.8, 18.2, and 28.4 months in patients with 0–1, 2, and 3–4 points, respectively. In the present study, the median survival was 9.6 months (291 days), 11.6 months (354 days), and 33.7 months (1023 days) in the three subgroups, respectively.

In the original publication of the revised Katagiri scoring system,^9^ the authors suggested a score of ≤3 for a survival rate > 80% at 12 months, 4–6 for a survival rate of 30–80%, and 7–10 for a survival rate ≤ 10%. In the present study, the survival rates at 12 months were 78.0%, 52.4%, and 21.6% in patients with scores of ≤3, 4–6, and 7–10, respectively.

In the original publication of the VDL score,^10^ the mean survival was 4.8, 13.1, and 18.3 months in patients with 0–3, 4–5, and 6 points, respectively. In the present study, the mean survival was 14.2 months (433 days), 20.0 months (608 days), and 28.4 months (864 days) in patients with 0–3, 4–5, and 6 points, respectively.

**FIGURE 2:**
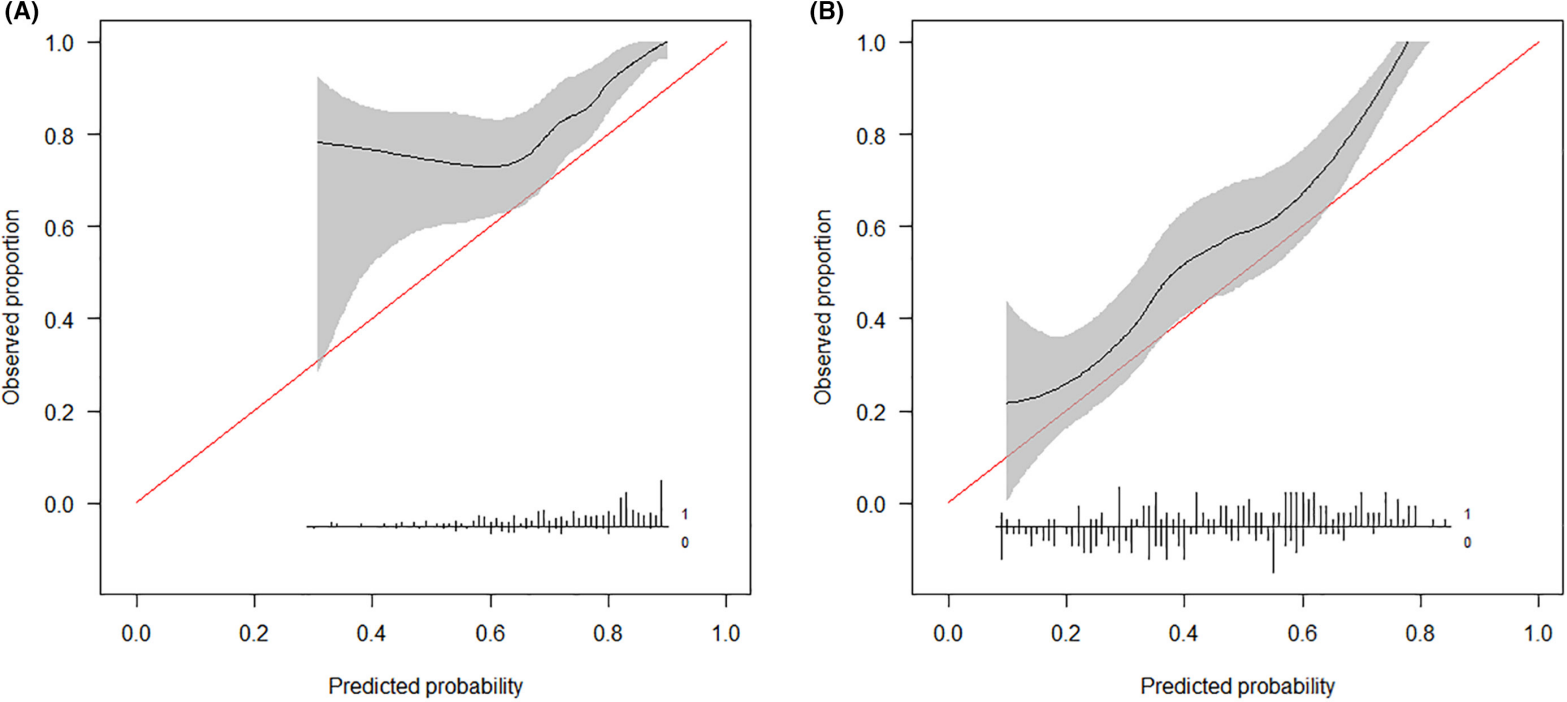
Calibration plots for 90‐day (A) and 1‐year (B) survival prediction using the SORG nomogram in the validation cohort, *n* = 268.

On using the SORG nomogram to predict 90‐day survival, the calibration curve suggested an obvious underestimation of survival, with an intercept of 0.821 and a slope of 1.093 (Table 5, Figure 2). Regarding the prediction of 1‐year survival, a slight underestimation was also observed, with an intercept of 0.476 and a slope of 1.150 (Table 5, Figure 2).

**TABLE 5:** Overview of the performance measures for 90‐day and 1‐year survival for the SORG nomogram and SORG ML.

Scoring system	Intercept		Slope		Brier score		Null‐model Brier score	
	90‐day survival	1‐year survival	90‐day survival	1‐year survival	90‐day survival	1‐year survival	90‐day survival	1‐year survival
SORG nomogram	0.821	0.476	1.093	1.150	0.128	0.211	0.116	0.247
SORG ML	1.242	0.118	0.500	0.733	0.141	0.193	0.116	0.247

Abbreviation: ML, Machine‐learning; SORG, Skeletal Oncology Research Group.

**FIGURE 3:**
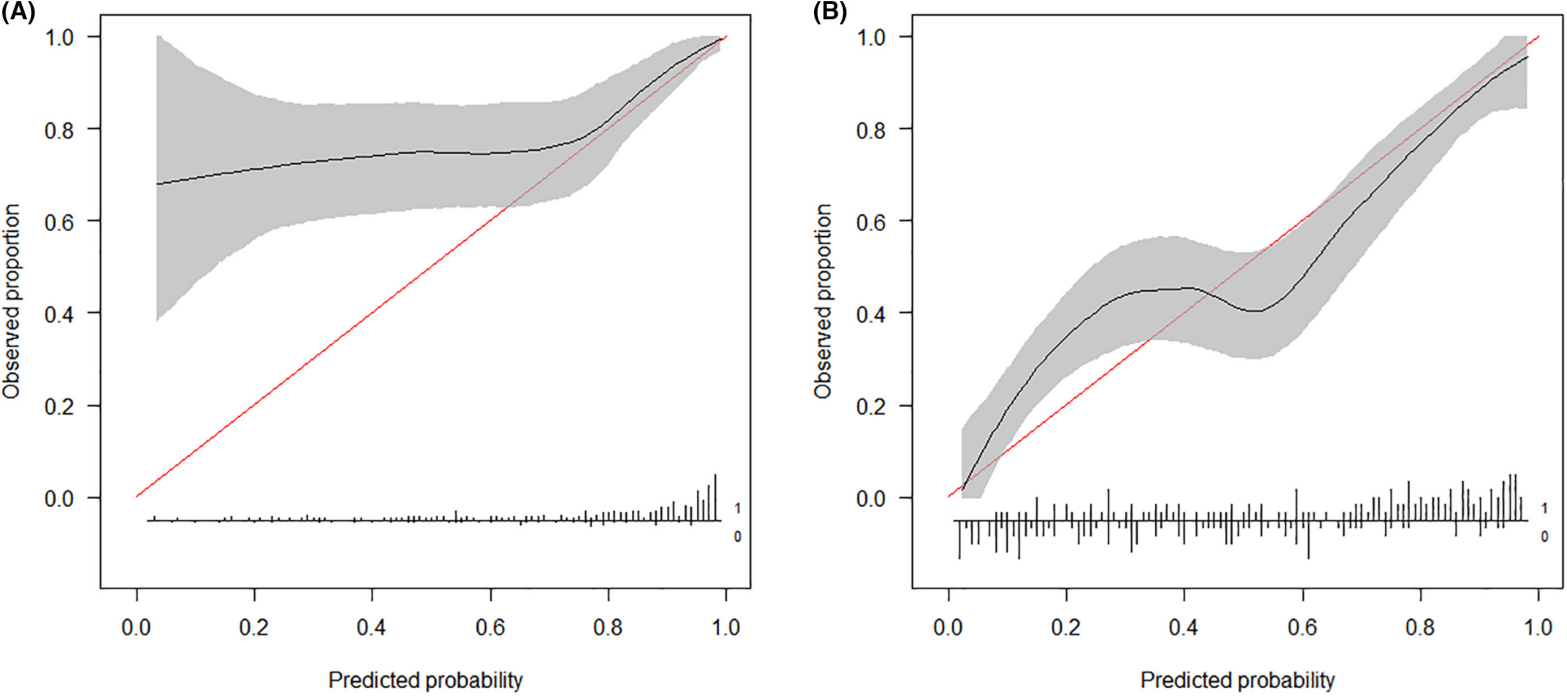
Calibration plots for 90‐day (A) and 1‐year (B) survival prediction using the SORG machine‐learning scoring system in the validation cohort, *n* = 268.

On predicting the 90‐day survival using the SORG ML scoring system, the calibration curve exhibited favorable calibration when the predicted probability exceeded 0.8. With predicted probabilities <0.8, the algorithm clearly underestimated the survival probability, accompanied by an overly extreme risk estimation in patients with 90‐day survival reflected by a positive intercept of 1.242 and slope of 0.500 (Table 5, Figure 3). On predicting 1‐year survival using the SORG ML scoring system, the calibration curve exhibited favorable calibration; slightly underestimated the survival rate when the predicted probability was <0.4; slightly overestimated the survival rate when the predicted probability exceeded 0.4, overall calibration intercept was 0.118, and overall calibration slope was 0.733 (Table 5, Figure 3).

## 4 DISCUSSION

Patients with symptomatic spinal metastases can benefit from surgery.^3^, ^22^, ^23^, ^24^ However, due to the high risk and cost, an accurate assessment of life expectancy is an important prerequisite for optimal treatment‐course determination and objective physician‐patient communication.^25^, ^26^, ^27^, ^28^, ^29^ Various prognostic scoring systems incorporating different variables with varying weights have been developed to predict the survival time of patients with spinal metastasis.^30^ However, few studies compared them, especially with more recent patient samples. With advances in medical oncology, especially targeted molecular treatments, long‐term survival is also possible for patients typically considered to have limited survival, challenging traditional surgical decision‐making aids.^31^ In addition, differences in health care policies, ethnicity, and culture render it imperative to externally validate the predictive models using samples from different populations.^13^, ^32^ To the best of our knowledge, no previous study has extensively compared different prognostic scoring systems in Chinese patients. In the present study, we evaluated the commonly used predictive scoring systems for spinal metastasis in a Chinese cohort from a single regional oncology center.

Leithner et al.^8^ evaluated the predictive value of seven preoperative prognostic scoring systems (the original and revised Tokuhashi, Sioutos, Tomita, VDL, Bauer, and modified Bauer scoring systems) and found that the original Bauer and the modified Bauer scoring systems exhibited the best association with survival, thus appearing to be highly predictive and practicable. Choi et al.^16^ assessed the clinical accuracy of six commonly cited prognostic scoring systems (the original Tokuhashi, Tomita, Bauer, VDL, Rades, and Bollen scoring systems) for patients with spinal metastases using a prospective multicenter cohort study. The study found that all scoring systems were capable of categorizing patients into separate prognostic groups with different overall survivals. However, none of the scores managed to achieve satisfactory concordance. Ahmed et al.^17^ compared the predictive ability of nine widespread scoring systems (the original Tokuhashi, revised Tokuhashi, Tomita, original Bauer, modified Bauer, Katagiri, VDL, SORG classic, and SORG nomogram) for patients undergoing surgical treatment for metastatic spine disease. They found that the SORG nomogram performed optimally in predicting 30‐day and 90‐day postoperative survival. The original Tokuhashi scoring system performed optimally in predicting 1‐year postoperative survival. Each scoring system's accuracy was influenced by primary‐tumor etiology and postoperative time point. Tabourel et al.^18^ sought to validate the prognostic accuracy of six preoperative scoring systems for spinal metastases, including the revised Tokuhashi, Tomita, modified Bauer, VDL, Rades, and Lei^33^ scoring systems. The study found that the AUC of the Tokuhashi score was the highest, and true patient survival was superior to that predicted using prognostic scores.

Overall, the SORG series exhibited superior predictive performance in the present study, possibly because it incorporates certain variables that other systems ignore, despite being highly predictive, including previous systemic therapy, laboratory data, and the distinction between brain metastases and those of other organs. Previous systemic therapy has been considered a reflection of a more developed cancer stage.^19^ Certain prognostically relevant laboratory data, including lymphocyte counts, hemoglobin concentration, and C‐reactive protein, reflect the nutritional status, reserve function of the organ systems, and inflammatory status of the body.^9^, ^12^, ^34^ Differentiation of the brain from other visceral metastases potentially contributes to an improved predictive accuracy because of the brain's unique cell types, anatomical structures, metabolic constraints, and immune environment.^35^, ^36^ Metastasis of the brain is associated with poorer survival outcomes and is clinically challenging. Although the SORG nomogram exhibited satisfactory discrimination, with an AUC of 0.722 for 90‐day survival prediction and 0.757 for 1‐year survival prediction, the calibration curves revealed certain unsatisfactory aspects of this model. First, in 90‐day survival prediction, only four patients (1.5%) had a predicted probability <0.4 in our series, influencing validation in this range. This uneven distribution of samples was also apparent in another study.^11^ Second, it obviously underestimated the actual survival probability of patients with lower predicted probability. Theoretically, the sophistication of machine‐learning predictive models increases predictive accuracy. In this study, the SORG ML scoring system also outperformed other models in terms of predictive performance; nonetheless, its advantage was not obvious. In other studies, the SORG ML scoring system has also proven to perform well in external validation; however, it does not measure up to that in the original publication,^13^, ^14^, ^15^ especially in diverse populations.^13^ Future research should be conducted to explore the causes of this inconsistency and how to improve the model to increase its external generalizability.

Almost all systems performed well in predicting breast cancer in the present study. This was probably because the pathological type of breast cancer itself is already a strong prognostic indicator of long‐term survival. The 1‐year survival for breast cancer was 76.9% in this study. According to our results, we also found that the short‐term predictive ability was superior to the long‐term predictive ability. The error may be attributed to the fact that many patients with advanced breast cancer have achieved long‐term survival, even though the traditional scoring system considered their survival time to be limited.^37^ With the survival improvement in metastatic lung cancer due to the introduction of new therapies, several scoring systems have demonstrated their insufficiency in predicting accuracy.^38^, ^39^ Among these scoring systems, only the revised Katagiri and SORG ML scoring systems have been able to distinguish lung cancer with sensi‐mutation and non‐sensi‐mutation; moreover, our results suggest that these scoring systems tend to be more accurate in lung cancer prediction.

Although incorporating more factors associated with treatment outcome may improve the accuracies of the predictive systems,^9^, ^12^, ^17^, ^19^ it also increases the impracticality. The SORG ML scoring system, in particular, also requires data input into specific digital applications. This limits its applicability in certain situations where a preliminary judgment must be made within a short period of time, such as an ambulatory consult, or when the patient evaluation is incomplete. Simple models with sufficient but not outstanding accuracy would be more practical and user friendly.^40^ In the present study, the modified Bauer and VDL scoring systems proved to have acceptable predictive performance, especially for breast cancer, despite incorporating fewer variables.

For the development of scoring systems, the original studies provided the reference survival time for each subgroup, based on their samples. In the present study, the actual survival time was underestimated in most scoring systems compared with the original reference survival time, especially in the subgroup considered to have a poor prognosis. The better survival in our series may be attributed to several factors. First, the patents included in this study were relatively recent, and more advanced comprehensive oncology treatments were administered. Second, the proportion of lung cancer patients in this study was 26.5%, and prediction systems tend to underestimate survival time in these patients.^41^ Third, Chinese patients, caregivers, and doctors tend to be more conservative toward advanced cancer,^42^ and several patients with surgical indications eventually opt for non‐surgical treatment due to a deteriorated physical condition or poor prognosis, which were inadequately reflected in the scoring systems.

In patients with spinal metastases, expected survival is an important determinant of surgical decision making. However, considering that the main purpose of surgical treatment is often symptom palliation, the impact of surgery on postoperative health‐related quality‐of‐life (HRQOL) outcomes should not be understudied.^18^, ^43^ A previous study that used HRQOL as the primary outcome challenged the traditional decision‐making process based on life expectancy.^31^ The development of a predictive model for postoperative HRQOL will be of great importance in the future.^43^

This study has certain limitations. First, except for lung and breast cancer, the sample size of other pathological types was insufficient to assess the scoring systems' capacity to predict such types. Second, the generalizability of our findings is limited since the study was performed at a single center. Although postoperative follow‐up is conducted prospectively, it must be acknowledged that retrospective data analyses for purposes other than the intended study have methodological limitations. Third, because the significance of expected survival evaluation differs between surgically and non‐surgically treated patients, only surgically treated patients were included in this study.

## 5 CONCLUSIONS

In conclusion, none of the scoring systems can perform optimally at all time points and for all pathology types. The reference survival times provided in the original study need to be updated. Moreover, a cautious awareness of the underestimation by these models is of paramount importance in relation to current patients.

## AUTHOR CONTRIBUTION

Weitao Yao designed and directed the project. Zhehuang Li, Liangyu Guo, and Bairu Guo were involved in the acquisition of data. Zhehuang Li analyzed the data and drafted the article. Peng Zhang, Jiaqiang Wang, and Xin Wang critically revised the manuscript. All authors reviewed the submitted version of the manuscript. Weitao Yao approved the final version of the manuscript on behalf of all authors.

## FUNDING INFORMATION

The study was funded by the Scientific Research Startup Fund of Affiliated Cancer Hospital of Zhengzhou University.

## ETHICAL APPROVAL STATEMENT

Our institutional review board approved a waiver of our study due to its retrospective nature.

## Data Availability

The data that support the findings of this study are available on request from the corresponding author. The data are not publicly available due to privacy or ethical restrictions.
